# Crystal structure of eveslogite revealed by electron diffraction techniques: a titanosilicate mineral with structurally and compositionally distinct nanoscopic tubular units

**DOI:** 10.1107/S2052520626008620

**Published:** 2026-09-01

**Authors:** Emilia Buchsteiner, Mariana Klementová, Wulf Depmeier, Sergey V. Krivovichev, Michael Czank, Marco Schowalter, Lukas Palatinus, Ira V. Rozhdestvenskaya, Viktor Yakovenchuk, Ute Kolb

**Affiliations:** a Institute of Applied Geosciences, Geomaterial Science, Technical University of Darmstadt, Darmstadt, Germany; b Department of Structure Analysis, Institute of Physics of the Czech Academy of Sciences, Prague, Czechia; c Institute of Geosciences, Kiel University, Kiel, Germany; d Nanomaterials Research Centre, Kola Science Centre, Russian Academy of Sciences, Apatity, Russia; e Department of Crystallography, Institute of Earth Sciences, St. Petersburg State University, St Petersburg, Russia; f Institute of Solid State Physics, University of Bremen, Bremen, Germany; g Centre for High-Resolution Electron Microscopy, Johannes Gutenberg University, Mainz, Germany; University of Antwerp, Belgium

**Keywords:** three-dimensional electron diffraction, titanosilicate, transmission electron microscopy, nanotubules, complex silicate

## Abstract

While silicate nanotubules are unknown in synthetic materials so far, naturally occurring minerals provide several examples of nanoscopic tubular structural building units based upon silicate oxyanions. The crystal structure of eveslogite, a chemically and structurally complex natural silicate mineral from Eveslogchorr mountain, Khibiny massif, Kola Peninsula, Russia, with an idealized sum formula of K_17.5_(Ba,Sr)_4_(Na,Ca)_40_[(Ti,Nb,Fe,Mn)_11_Si_62_O_179_(OH,F)_12_(O,OH)_13_](H_2_O) has been solved using modern electron crystallography techniques. This study reveals the first example of a binary structure formed by two different types of transition-metal-modified silicate nanotubular units in a fibrous crystal. One of these is closely related to the nanorod occurring in the unitary nanotubule-based structure of yuksporite, whereas the other is unprecedented and can be considered as an (Nb, Ti)-modified derivative of the [Si_12_O_30_]^12−^ nanotubular units occurring in the binary all-silicate nanotubule-based structures of charoite polytypes. The eveslogite tubular units are packed in a tight arrangement and linked with each other through secondary inter­actions involving Ca^2+^- and Na^+^-centered polyhedra. The interiors and walls of the tubules are occupied by K^+^ and Ba^2+^ cations as well as H_2_O molecules. Topological analysis of the interpolyhedral connectivity shows that the walls of the nanotubules possess topologies related to those of lamprophyllite and delhayelite-group minerals. This topological relationship may indicate possible structural pathways linking these framework types and highlights interesting similarities of exfoliation processes that are well known in modern soft chemistry nanotechnologies. Information-based structural complexity calculations place eveslogite among the most complex minerals known so far. Our study further demonstrates the enormous potential of mineralogy to discover structures unprecedented among the currently known synthetic materials and may serve as an inspiration for the preparation of novel types of artificial nano­structures and their possible technical applications in various fields.

## Abstract 2

The study of the mineral eveslogite reveals a unique structure based upon two types of nanoscopic heterosilicate-based tubules.

## 1. Introduction

The discovery of free-standing carbon nanotubes in 1991 has been one of the milestones in the current development of nanoscience and nanotechnology (Iijima, 1991 ▸). This remarkable achievement was followed by the fabrication of various inorganic nanotubes, most prominently sulfide-based examples (Tremel, 1999 ▸; Tenne, 2002 ▸; Serra *et al.*, 2019 ▸), and by the recognition that free-standing nanotubes and nanotubular arrangements also occur in minerals including chrysotile, halloysite, imogolite, tochilinite, and many more (Krivovichev, 2008 ▸; Pasbakhsh & Churchman, 2015 ▸). The advent of modern electron crystallography methods, in particular high-angle annular darkfield (HAADF) imaging, high-resolution transmission electron microscopy (HRTEM), precession electron diffraction (PED) and three-dimensional electron diffraction (3D ED) (Kolb *et al.*, 2007 ▸; Kolb *et al.*, 2008 ▸; Kolb *et al.*, 2011 ▸; Mugnaioli *et al.*, 2009 ▸; Gemmi *et al.*, 2019 ▸; Simoncic *et al.*, 2023 ▸) allow us to reveal even more prominent nanoscopic tubular structural units in charoite (Rozhdestvenskaya *et al.*, 2010 ▸; Rozhdestvenskaya *et al.*, 2011 ▸) and denisovite (Rozhdestvenskaya *et al.*, 2017 ▸). In this paper, the term ‘nanotubule’ is used in a strictly structural sense for a one-dimensional polyhedral building unit with a cross section > 1 nm, whose wall encloses an axial cavity and is more strongly connected internally than to neighboring units [see *e.g.* Adelani (2025 ▸), and references therein]. The term does not imply an isolated or open-ended hollow fiber and it does not describe the external morphology of eveslogite, which is fibrous. The application of synchrotron radiation techniques to the crystal structure of yuksporite, a unique titanosilicate from the Khibiny alkaline massif on the Kola peninsula, Russia, led to the discovery of the first titanosilicate nanotubules (or nanorods) that had no precedents in both natural or synthetic materials (Krivovichev *et al.*, 2004 ▸). It is noteworthy that silicate and titanosilicate minerals with nanotubular arrangements are unknown in current synthetic chemistry, but occur in nature in large (sometimes hundreds of kg) quantities in various geological settings such as the Khibiny massif on the Kola peninsula (denisovite, yuksporite) or the Murun massif in Yakutia (charoite, denisovite). Herein we report on yet another, so far only fragmentarily elucidated, nanotubule-based crystal structure of eveslogite, a complex Ca–Na–K–Ba–Sr–Ti–Nb–Fe–Mn silicate from Eveslogchorr mountain located in the Russian Arctic.

For the sake of easier readability, the most frequently used mineral names eveslogite, yuksporite, charoite and denisovite will be denoted by their International Mineralogical Association–Commission on New Minerals, Nomenclature and Classification (IMA–CNMNC) approved mineral symbols (abbreviations) **Evl**, **Yks**, **Cha** and **Dnv**, respectively (Warr, 2021 ▸).

In contrast to the earlier discoveries of nanotubules in minerals, **Evl** is absolutely unique, as it contains two types of (Ti, Nb, Fe, Mn)-modified silicate tubules and therefore can be considered as a binary nanotubule-based heterosilicate structure (or titanosilicate structure because of its overall Ti-dominance). The application of multiple modern electron crystallography techniques allowed us to reveal its amazing structural arrangement and to suggest possible mechanisms of its formation in nature through exfoliation processes, well established in the current fabrication methods of nanosheets and nanotubes from layered starting materials.

The mineral **Evl** has its type locality at Eveslogchorr mountain, Fersman-Gorge, Khibiny, Kola Peninsula, Russia. According to the present knowledge, this is the only occurrence of eveslogite. By external characteristics, the mineral is practically indistinguishable from **Yks** and forms fibrous aggregates of thin, flexible needle-like crystals [Fig. 1 ▸(*a*)]. It should be noted that **Yks** occurs not only at its type locality, the nearby Yukspor mountain, but also at various other places in the extensive rischorrite (poikilitic nepheline syenite) zone of the Khibiny massif, including Eveslogchorr mountain. It also occurs in the alkali pluton of the Murun Massif (Sakha, Yakutia, Russia) (Konev *et al.*, 1985 ▸), where the related minerals **Cha** (Rogova *et al.*, 1978 ▸) and **Dnv** (Men’shikov, 1984 ▸; Konev *et al.*, 1987 ▸) were found as well. **Yks** is thus a relatively widespread mineral. Probably because of its relative abundance **Yks** had already been discovered in 1923 by E. E. Kostyleva (Fersman, 1923 ▸; Yakovenchuk *et al.*, 2005 ▸), while the rare **Evl** was described only in 2003 (Men’shikov *et al.*, 2003 ▸). It was consistently reported that **Evl** occurs as a late-hydro­thermal formation in veins breaching the aforementioned poikilitic nepheline syenite at Eveslogchorr mountain. A similar situation applies to **Yks**.

The structure of both minerals could not initially be solved with the methods and instruments of X-ray diffraction analysis known and available at the time of their discoveries and initial description. Around the turn of the millennium, G. Ferraris and colleagues developed an approach that sought to derive model ideas for complex heterophyllosilicate structures from known metric relationships of modular building units (Ferraris & Gula, 2005 ▸). These authors suggested that both **Yks** and **Evl** were heterophyllosilicate structures of the astrophyllite family, *i.e.* based upon sheets of Ti octahedra (or semi-octahedra) and chains of Si tetrahedra. The solution of the crystal structure of **Yks** by means of synchrotron diffraction (Krivovichev *et al.*, 2004 ▸) made it clear that it does not belong to the so-called heterophyllosilicate structures, but is composed of nanotubular or nanorod building units (see also the review on tubular chains in the structures of natural and synthetic silicates (Rozhdestvenskaya & Krivovichev, 2011 ▸). For **Evl**, the time for a final structure analysis had not yet come, however, doubts about the applicability of the modular approach to this case were increasing. In a comprehensive work by Ferraris & Gula (2005 ▸) the authors refer in a short paragraph about the modular model for the **Evl** structure (based on astrophyllite) to a personal communication of N. V. Chukanov, who had observed similarities between the IR spectra of **Evl** and **Yks**. This observation put the astrophyllite-based model by Men’shikov *et al.* (2003 ▸) in doubt, without having already provided a new improved model. All attempts to solve the structure of **Evl** by now conventional synchtrotron-radiation-based diffraction methods did not succeed. Intermediate successes in structure determination of similarly complex silicate structures occurring under closely related geochemical conditions using 3D ED (Rozhdestvenskaya *et al.*, 2010 ▸; Rozhdestvenskaya *et al.*, 2011 ▸) were encouragements to attempt structure solution of **Evl** using similar approaches. Below we show that, even if the suggestions made in (Men’shikov *et al.*, 2003 ▸) about the nature of **Yks** and **Evl** did not match exactly, there is a certain degree of relationship between them and heterophyllosilicates in terms of imaginary exfoliation procedures.

## 2. Results

### 2.1. Data collection, unit-cell and space group determination

Many attempts were made to collect X-ray diffraction data on the **Evl** crystals using both in-house and synchrotron radiation sources. All the attempts failed due to the small size of the crystals, their intergrowth and/or twinning and their curved and fibrous morphology. 3D ED experiments were therefore essential. Many crystals (>50) were tested using the method of fast automated diffraction tomography (Fast-ADT) (Plana-Ruiz *et al.*, 2020 ▸) and with PED on two different transmission electron microscopes (see *Methods*[Sec sec4.3] for details). Most datasets came from twinned crystals, but a few corresponded to untwinned crystals. The two best untwinned datasets [Figs. 1 ▸(*b*)–1 ▸(*i*)] were combined into a final dataset for subsequent structure analysis.

In the original publication (Men’shikov *et al.*, 2003 ▸), based on powder diffraction data, the assigned space group was *P*2/*m*, with lattice parameters (after transformation from the monoclinic *B*-setting): *a* = 14.069 (3) Å, *b* = 44.31 (2) Å, *c* = 24.937 (5) Å, β = 95.02 (4)°, *V* = 15486 (13) Å^3^. In variance with this estimate, our 3D ED data yielded a monoclinic cell with the parameters: *a* = 14.1898 (9) Å, *b* = 44.7704 (1) Å, *c* = 15.9111 (18) Å, β = 109.468 (4)°. This unit cell was used to fit the powder diffraction data. The Le Bail fit (Fig. S1) showed that this cell describes the bulk material very well. The unit cell refined against the powder diffraction data differed only slightly from the 3D ED cell, namely: *a* = 14.2358 (2) Å, *b* = 44.8239 (5) Å, *c* = 15.9058 (4) Å, β = 109.658 (2)°. As powder diffraction data usually yield more accurate unit-cell parameters than 3D ED data, we decided to use these cell parameters for subsequent analysis. The difference between our cell parameters and those reported in (Men’shikov *et al.*, 2003 ▸) may cast doubt if our sample is really **Evl**. However, the identity of the material studied in this work as **Evl** is ensured by the use of the holotype sample and by the good agreement of the X-ray powder diffraction pattern of the sample (Fig. S1) with that reported by Men’shikov *et al.* (2003 ▸). The discrepancy in unit-cell parameters can be explained by the severe twinning that is characteristic for **Evl**. In twinned crystals, reflections from the two twin domains overlap and obscure the true lattice, making unit cell determination highly challenging. We found that diffraction data from twinned crystals can be indexed using a cell very similar to that reported in the original work, whereas analysis of a rare untwinned crystal revealed the true unit cell.

The reflection conditions extracted from the data were 0*k*0: *k* = 2*n*. Reflections of the type *h*0*l*: *h* = 2*n*+1 were much weaker than reflections with *h* = 2*n* in the *h*0*l* plane, indicating a possible presence of an *a* glide. However, reflections with *h* = 2*n*+1 are weaker than *h* = 2*n* in the entire dataset due to the presence of a twofold superstructure along *a*. A careful evaluation of the reflection intensities led us to discard the possibility of an *a* glide in the structure of **Evl**. Hence, the two possible space groups consistent with the observed reflection conditions were *P*2_1_ and *P*2_1_/*m*. The structure solution (see below) showed unequivocally that the mirror plane is not present in the structure, and the correct space group for **Evl** is thus *P*2_1_.

### 2.2. Structure analysis

Structure determination for **Evl** was performed with *JANA2020* (Petříček *et al.*, 2023 ▸) based on datasets evaluated by *PETS2* (Palatinus *et al.*, 2019 ▸) that showed no twinning. The details of the structure determination and refinement are summarized in Table 1 ▸. The structure was solved by the charge flipping algorithm (Palatinus, 2013 ▸) using the program *SUPERFLIP* (Palatinus & Chapuis, 2007 ▸). The solution was checked for the presence of symmetry elements and the presence of a mirror plane in the structure could be excluded. The space group *P*2_1_ was confirmed by the analysis of the solution. The structure refinement was performed using the kinematical theory of diffraction (kinematical refinement), and converged at a residual *wR*(all) value of 28.54%. Due to the complexity of the structure and the kinematical approximation used in the refinement, restrictions had to be applied to stabilize the structure model. All ADPs were refined isotropically and constrained to be equal for atoms of the same species. They converged at reasonable values with the K positions having the highest *U*_iso_ values of 0.0223 Å^2^. Si–O bond lengths were restrained to a target value of 1.61 Å using a restraint standard uncertainty σ = 0.01 Å in *JANA2020*. This σ controls the restraint weight and is not the standard deviation of the refined Si–O bond length distribution.

Fig. 2 ▸(*a*) shows the resulting crystal structure of **Evl** in projection along the *a* axis. The unit cell contains 345 independent atom positions. The skeleton of the structure consists of 62 SiO_4_ tetrahedra in the asymmetric unit, which condense into three-periodic extended silicate chains and fold up to form two different kinds of tubules, designated **Yk** (yuksporite-like tubule) and **Ch** (charoite-like tubule), running parallel to [100] [Fig. 2 ▸(*b*)]. Adjacent to the tubules, there are 18 polyhedra, four of which contain highly scattering species that were attributed to mixed-occupancy Ba/Sr sites and the remaining fourteen were interpreted as K atoms. The occupancies of the Ba/Sr positions were freely refined. Additionally, seven K atoms at 0.5 occupancy were located in the interior of the tubules. Two octahedra of the **Ch** tubule are occupied by Ti/Nb. The refinement of their occupancies showed a slight preference for Nb on both sites. The other tubule, **Yk**, contains eight (Ti,Nb,Fe,Mn)O_6_ octahedra, which are referred to as MO_6_ in the following. The occupancies of the M positions could not be refined reliably. The ratio of metal ions in these positions was therefore fixed according to the average chemical composition estimated from multiple energy-dispersive X-ray spectoscopy (EDS) analyses. These EDS measurements should be regarded as semi-quantitative and provide only an estimate of the average cation ratios present in the material. Nevertheless, in the absence of a stable refinement of these occupancies and given the lack of alternative site-specific chemical information, the average EDS composition provided the most chemically reasonable basis for constructing the structure model. After correction for the Nb/Ti occupancy in the octahedra of the **Ch** tubule, leading to an occupancy for each M position of 0.740, 0.048, 0.134 and 0.079 for Ti, Nb, Fe and Mn, respectively. A similar procedure was followed for the (Fe,Mn)O_5_ square pyramid in the interior of the **Yk** tubule, where the occupancies were set to 0.63 and 0.37 for Fe and Mn, respectively, based on the average semi-quantitative chemical data. The **Yk** and **Ch** tubules are interconnected by 40 independent (Ca,Na)O_*x*_ polyhedra that form ribbons parallel to [100]. The occupancies of Ca and Na in these polyhedra could be refined. In 14 positions the refinement indicated a position fully occupied by Ca, three positions refined to full occupation by Na. Overall, 30 (Ca,Na)O_*x*_ polyhedra are Ca dominant and ten Na dominant.

The complexity of the **Evl** structure necessitated the use of several constraints, restraints and chemically informed assumptions during refinement. A summary of the refinement strategy is provided in Table 2 ▸. While all atomic positions were refined against the diffraction data, several occupancies and displacement parameters required additional constraints to obtain a chemically reasonable and stable model. Detailed bond lengths, mixed site occupancies and hydrogen placement should therefore be interpreted cautiously.

The kinematical refinement converged at a residual *wR*(all) value of 28.54%. Although this value is higher than typically reported for 3D ED structures, it should be considered in the context of the exceptional complexity of the structure. The unit cell contains 345 independent atom positions with a volume of 9558.1 Å^3^, making full dynamical refinement computationally prohibitive. Consequently, no dynamical refinement was attempted, as the required computational resources and calculation times were beyond practical limits for a structure of this size and complexity. Furthermore, EDS analyses revealed significant compositional variations between individual crystals (see Section 2.4). The refined model therefore represents an average structure with average site occupancies rather than the exact local composition of any single crystal. Despite the relatively high *wR*(all), the refined structure is consistent with the features observed in the HAADF STEM images (see Section 2.5) and provides a satisfactory description of the powder diffraction data, as demonstrated by the Le Bail fit (Fig. S1).

### 2.3. Major structural details of the Evl structure

Two of the essential building blocks that make up the **Evl** structure are heterosilicate tubules of the two types, **Yk** and **Ch**, oriented parallel to the *a* axis and occurring in a 1:1 ratio. The dominant heteroatom in the **Yk** tubules is Ti, while the **Ch** tubules are moderately Nb dominant. It should be noted that the analyses show that the Nb content seems to vary significantly from one crystal to another and therefore the results should be considered with due caution. The packing of the tubules is shown schematically in Fig. 2 ▸(*c*). The tubules are linked through octahedrally coordinated Ca^2+^ and Na^+^cations located in-between them. The same general structural building principle is realized in the related minerals **Cha**, **Dnv** and **Yks**.

An essential building unit of both types of tubules are double or triple *dreier* silicate chains (that is, with three corner-sharing SiO_4_ tetrahedra within their identity periods (Liebau, 1985 ▸), containing branches. The periodicity of the chains expresses itself in the lattice parameter of ∼7.1 Å, or ∼14.2 Å if subject to the formation of a superstructure.

The charoite-like tubules **Ch** [Figs. 3 ▸(*a*), 3 ▸(*b*), 3 ▸(*g*)] of **Evl** differ from those observed in **Cha** (Rozhdestvenskaya *et al.*, 2010 ▸; Rozhdestvenskaya *et al.*, 2011 ▸) and **Dnv** (Rozhdestvenskaya *et al.*, 2017 ▸) [Figs. 3 ▸(*c*), 3 ▸(*d*), 3 ▸(*h*)] essentially. The **Ch** tubule in **Evl** can be obtained from that of **Cha** by replacing every third SiO_4_ tetrahedron in one of the *dreier* silicate chains by a NbO_6_ octahedron and attaching external [Si_2_O_7_]^6−^ groups that link each NbO_6_ octahedron with its respective neighbors along the chain extension. The **Ch** tubule thus obtains a pear-shaped cross section with outer dimensions of about 1.2 nm × 1.4 nm. Similar to zeolites (Yu *et al.*, 2024 ▸), the free inside dimensions of the tubule can be estimated from the nearest non-bonded O⋯O distances across the internal space of the tubule minus 2 × *r*(O^2−^) adding up to ∼2.8 × 2.0 Å^2^. This is large enough to accommodate K^+^ and Na^+^ cations, or maybe even allow for the possibility of ionic exchange reactions. The chemical composition of **Ch** can be expressed as [(*M*O)(Si_2_φ_7_)(Si_11_φ_29_)], where φ = O, OH and *M* = Nb, Ti. Despite the evident similarity of the **Ch** tubule with the corresponding basic ones in **Cha** and **Dnv**, it is a new structural motif, which has not been observed previously in any mineral or synthetic compound.

The **Yk** tubule in **Evl** [Figs. 3 ▸(*e*), 3 ▸(*f*), 3 ▸(*i*)] is topologically concordant to the tubule found in **Yks** (Krivovichev *et al.*, 2004 ▸) and also has an elliptical cross section. It is confined by two bow-shaped unbranched dreier double silicate chains at the opposite ends of the long diameter of the ellipse. The area in between is marked by a stacking of *M*O_6_ octahedra (*M* = Ti^4+^, Nb^5+^, Fe^3+^, Mn^2+^, see above), which are located at the corners of a cube-like orthorhombic prism (‘M-cube’) [Figs. 3 ▸(*e*), 3 ▸(*f*), 3 ▸(*i*)]. The MO_6_ octahedra are interconnected by two differently oriented symmetrically independent [Si_2_O_7_]^6−^ diorthosilicate groups. The ‘vertical’ groups are oriented parallel to the tube axis and located at the opposite ends of the short diameter of the ellipsis. These groups are connected with *M*O_6_ octahedra only and lie at the exterior of the **Yk** tubule. The [Si_2_O_7_]^6−^ groups of the second kind are ‘horizontal’ and wrapped in the interior of the nanotubules parallel to the short axis of the ellipse. Along the tube axis, two consecutive horizontal [Si_2_O_7_]^6−^ groups have alternating up and down orientations of their tetrahedra. Their apical oxygens face each other, leading to the formation of a square-planar coordination around a (Fe,Mn) cation at the center of the cross section. An additional oxygen roughly perpendicular to the square completes the square-pyramidal coordination around (Fe,Mn). The (Fe,Mn)–O_apical_ (possibly H_2_O, see below) vector determines the polarity in the polar space group *P*2_1_ of **Evl**. The up and down inward orientations of the horizontal [Si_2_O_7_]^6−^ in a given M-cube impose reversed order of the up and down orientations of the tetrahedra in the adjacent M-cubes. As a result, these M-cubes cannot contain (Fe,Mn)O_5_ groups, but remain empty. This arrangement is the crucial reason for the formation of the superstructure with doubled lattice parameter *a* in **Evl** (14.2359 Å) compared to that of **Yks** (7.1260 Å), where all horizontal [Si_2_O_7_]^6−^ groups have the same arrangement of ups and downs in their respective M-cubes. The (Fe,Mn) positions in the filled M-cubes of **Evl** are fully occupied and the unfilled ones are empty, whereas all the M-cubes of **Yks** are evenly filled, albeit with reduced occupancy. The outer dimensions of the **Yk** tubule are ∼1.8 nm × 1.5 nm, whereas its two internal compartments have their crystallographic free diameters of ∼2.9 Å × 4.5 Å, which allows for the accommodation of K^+^ cations.

The binary nanotubule-based structure observed for **Evl** is unprecedented among any known minerals or synthetic inorganic compounds.

### 2.4. Chemical composition and crystal chemical formula

EDS measurements of **Evl** crystals were performed at the TEMs as well as employing electron microprobe analysis (EMPA). These methods provide semi-quantitative compositions. The mineral contains a large variety of elements, mainly Si, Ca, Na, K, Ti but also to a lesser extent Sr, Ba, Fe, Nb and Mn (Table 3 ▸). Some crystals contained small amounts of F and Cl as well. Oxygen is an essential framework constituent by stoichiometry, whereas hydrogen could not be measured directly. The compositional range for different crystals was rather large with the Ca content ranging from 24.1 to 37.4 atoms per formula unit (apfu), the Na content from 10.0 to 22.1, the K content from 12.3 to 21.1, and the F content ranging from 0.0 to 11.3 apfu (Table 3 ▸). Other elements like Sr or Fe, however, showed only little variation. Where site occupancies could not be stably refined during structure analysis, the average chemical data were used. The empirical composition normalized to Si = 62 can be written as K_17.50_Ba_1.85_Sr_2.15_Na_11.91_Ca_28.09_Ti_6.70_Nb_1.60_Fe_1.70_Mn_1.00_Si_62_O_200_F_5_H_*n*_ and the structure based idealized formula is K_17.5_(Ba,Sr)_4_(Na,Ca)_40_[(Ti,Nb,Fe,Mn)_11_Si_62_O_179_(OH,F)_12_(O,OH)_13_](H_2_O). Because H and the detailed O/OH/F partitioning were not measured directly, the latter should be regarded as a model formula. The empirical chemical formula corresponds to the calculated density of 2.84 g cm^−3^. The original IMA formula (Men’shikov *et al.*, 2003 ▸) was (Ca,K,Na,Sr,Ba)_24_[(Ti,Nb,Fe,Mn)_6_­(OH)_6_Si_24_O_72_](F,OH,Cl)_7_. The re-calculation of the chemical data given in the original study of **Evl** (24) to Si + Al = 62 results in the empirical formula K_15.91_Rb_0.21_Ba_1.65_Sr_2.33_Na_13.02_Ca_29.19_Ti_7.18_Nb_4.34_Fe_1.34_Mn_1.24_Zr_0.26_Ta_0.10_(Si_61.43_Al_0.57_)O_180.01_(OH)_26.66_F_12.60_Cl_1.03_. Grouping comparable site populations provides (K,Rb)_16.12_(Ba,Sr)_3.98_(Na,Ca)_42.21_(Ti,Nb,Fe,Mn,Zr,Ta)_14.46_(Si,Al)_62_O_180.01_(OH)_26.66_F_12.60_Cl_1.03_. The comparison of the two formulas shows good agreement for the major cation populations, particularly the (Ba,Sr) and (Na,Ca) groups, whereas larger differences are observed for the transition metals and the volatile anions (F, OH, Cl). These differences may reflect crystal-to-crystal heterogeneity, differences in analytical methodology and the limited ability to directly constrain H/OH/F partitioning. Nevertheless, the overall chemical characteristics are comparable and both studies yield very similar calculated densities of 2.85 g cm^−3^ and 2.84 g cm^−3^, respectively. Thus, despite differences in some minor constituents, the present chemical data are broadly consistent with the original description of **Evl**.

### 2.5. HAADF imaging

The **Evl** structure model was compared with a filtered HAADF image. The image is oriented along [100] and shows the different tubules [Fig. 4 ▸(*a*)]. By superimposing the model onto the HAADF image it can be seen that the atom positions agree very well [Fig. 4 ▸(*b*)]. Minor discrepancies in atom positions could be attributed to a slight sample mis-tilt relative to the electron beam during the acquisition of the image. To compare the atom species, HAADF simulations were performed with the obtained structure model [Fig. 4 ▸(*d*)]. They agree well with the intensity distribution of the HAADF image [Fig. 4 ▸(*c*)]. The bright cross-like structure that can be seen in the HAADF is caused by the M-cube and another bright spot located close to the tubular structure is caused by a (Ti,Nb) position. Minor discrepancies remain for some of the Ca/Na columns, for example, the Ca-dominated columns linking the **Yk** and **Ch** tubules, which appear slightly weaker in the experimental HAADF image than in the corresponding simulation. In the structure model, these positions were refined as fully occupied Ca sites and therefore contribute comparatively strongly to the simulated image. Because the HAADF image and the 3D ED datasets were acquired from different crystals, local enrichment of Na at these sites cannot be excluded. Such partial Ca/Na substitution would reduce the observed intensity in the simulated image.

### 2.6. Provisional H, OH, F and H_2_O assignments

Due to the inherent limitations of the data quality, it was not possible to determine hydrogen positions directly from the diffraction data. Furthermore, no complementary FTIR, Raman or EELS measurements were available to provide additional information on the distribution of F and OH groups. While such techniques may provide evidence for the presence of hydroxyl groups, they generally do not permit an unambiguous assignment of H atoms to specific crystallographic sites in a structure of this complexity. Moreover, as compositional variations were observed along individual crystals, corresponding variations in the distribution of H and F are also likely. Therefore, the possible protonation of O sites was evaluated on the basis of crystal chemical arguments. In silicate structures, O atoms that bridge two tetrahedrally coordinated cations (*e.g.* Si–O–Si) or a tetrahedral and octahedral cation (Si–O–*M*) generally function as framework-linking anions and are typically fully saturated within the structure. Protonation of such bridging O atoms would lead to unfavorable local bonding environments and is therefore was considered unlikely. Consequently, the 66 O atoms bridging two Si sites and the 53 O atoms bridging Si and *M* sites were excluded as potential OH groups. A similar argument applies to the 42 terminal O atoms of SiO_4_ tetrahedra, which are additionally bonded to three Ca/Na sites, as well as ten terminal O atoms of MO_6_ octahedra that are not bonded to Si. These O atoms already receive sufficient bond-valence contributions from surrounding cations and are therefore unlikely candidates for protonation. The most plausible protonation sites are O atoms with relatively low bond-valence requirements that are coordinated only by Ca/Na polyhedra or by a limited number of framework cations. These include 12 O atoms bonded exclusively to three Ca/Na sites and thirteen apical O atoms of SiO_4_ tetrahedra coordinated by two Ca/Na polyhedra. Such environments can readily accommodate OH groups without introducing unreasonable local bonding geometries. In addition, the apical O atom of the (Fe,Mn)O_5_ square pyramid inside the **Yk** tubule has only one strong bond to the central cation and can accommodate two H atoms. This site is therefore interpreted most plausibly as an H_2_O molecule. Based on the refined occupancies, the crystal structure of **Evl** has a charge deficit of −26.5 if all anion sites are assumed to be O^2−^. The 26 candidate protonation sites identified above compensate this deficit almost exactly when occupied by H. A further consideration concerns F incorporation. Five of the candidate anion sites coordinated only by Ca/Na polyhedra are located in environments where hydrogen bonding is geometrically unfavorable because of the close proximity of surrounding cations. These sites therefore represent the most plausible locations for F^−^ rather than (OH)^−^ anions. Thus, the proposed distribution of O, OH, F and H_2_O derived from the crystal chemical analysis of the structure model combined with the determined chemical composition shows that the model is compatible with the requirement of electroneutrality. Bond valence calculations were performed for the refined structure (Table S1), but they did not provide sufficient discrimination between alternative O/OH/F assignments and were therefore used only as a consistency check. This observation further strengthens the reliability and consistency of the refined structure.

### 2.7. Twinning

During the data evaluation, it was noted that besides a couple of single crystal datasets [Figs. 5 ▸(*a*),  ▸5(*b*)], a significant number of datasets showed a distribution of intensities that could not be reasonably explained by a single reciprocal lattice [Figs. 5 ▸(*d*), 5 ▸(*e*)]. The distribution of the spots was symptomatic of twinning. Indeed, the diffraction spots could all be indexed by two identical cells with different orientation matrices. The twinning is visible in the a*c* plane. The a* vector points to two directions, while the b* and c* vectors of the two lattices overlap. The reflections of the two lattices overlap when *h* = 4*n*. The twinning can be described by the twinning matrix [1 0 0 / 0 1 0 / 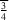 0 1]. The twin domains are related by a 180° rotation about the *a* axis, corresponding to a simple rotational twin law in the monoclinic system. The matrix for a twofold rotation about *a* is given by [1 0 0 / 0 1 0 / (2*c*cosβ)/*a* 0 1] (Koch, 2006 ▸). The use of the determined unit-cell parameters leads to the value of −0.747 for the (2*c*cosβ)/*a* term, which matches closely with the extracted matrix, confirming the rotation as a plausible twinning mechanism. It should be noted that a mirror plane as an alternative twinning operator would result in an equivalent diffraction pattern. Nevertheless, in real space, these two operations are not equivalent, as the structure lacks an inversion center. Structural modeling showed that the rotated twin produced a slightly better match at the interface and therefore only this twinning mode is described in the following. Nevertheless, the possibility of mirror-related twinning cannot be excluded.

In the structure, the twinning is realized as planar stacking faults, as documented by HRTEM (Fig. S2). The structural nature of twinning can be understood, taking into account polarity of both type of nanotubules. In the **Ch** nanotubule, the NbO_6_ octahedra have five of their vertices shared with SiO_4_ tetrahedra, whereas the sixth vertex is pending. The respective Nb-O bond is oriented outward with respect to the tubule. For the **Yk** tubule, it was mentioned above that its polarity is determined by the presence of the Fe–H_2_O bond in the tubule interior. Fig. 5 ▸(*c*) shows the scheme of packing of tubular building units in eveslogite with orientations of the Fe–H_2_O and Nb–O vectors. Assuming that the twinning operator is the twofold rotation around the *a* axis, it is easy to recognize that the twinning operation changes the orientations of the vectors into opposite directions [Fig. 5 ▸(*f*)]. Thus, the twinning involves rotation of tubules around their axes and formation of domains with different polarity directions. For twinning by a mirror plane, the polarity would be preserved.

## 3. Discussion

The determined structure of **Evl** revealed that it belongs to chain-, ribbon- and tube-silicates (Day & Hawthorne, 2020 ▸), sharing similarities with **Yks**, **Cha** and **Dnv**. The successful structure elucidation suspends earlier conjectures (Men’shikov *et al.*, 2003 ▸; Ferraris & Gula, 2005 ▸) that **Evl** might be a heterophyllosilicate of the astrophyllite-type, that is, a silicate based upon layers of SiO_4_ tetrahedra and MO_6_ octahedra. Our findings rather support an idea expressed in (Day & Hawthorne, 2020 ▸) that the structure of **Evl** bears a certain similarity with that of **Yks**, however, only up to a certain point, since the structure of **Evl** contains **Ch** in addition to **Yk** tubules. This unique architecture (binary nanotubule-based heterosilicate structure) may have its roots in the genetic mode of the mineral that may involve secondary crystallization from colloidal solutions formed due to the alteration of primary silicate minerals such as apophyllite, astrophyllite and lamprophyllite. The relation of **Evl** (and **Yks**) to the latter minerals can be demonstrated by the topological analysis of the tubules and layers using their nodal representation. Within this approach, each coordination polyhedron is symbolized (replaced) by the node, and the adjacent nodes are linked by an edge if the respective polyhedra share a common anion (Krivovichev, 2005 ▸). The nodal representations of nanotubules in **Evl** and **Cha** are shown in Fig. 6 ▸.

In order to get an idea of the underlying topology of the **Ch** tubule in **Evl**, it can be cut open along the tube axis [Fig. 6 ▸(*b*)] and unfolded into a plane resulting in a band [Fig. 6 ▸(*e*)], which is periodic along the tube axis but of finite width across the band. There are six four-membered rings and six eight-membered rings in the repeat unit (4^6^8^6^). Additionally, there are extended chains of six-membered rings (62) that consist of two opposed M octahedra, two [Si_2_O_7_]^6−^ groups and two SiO_4_ tetrahedra of the adjacent dreier triple chain of the (4^6^8^6^) unit. The (4^6^8^6^) and (6^2^) sub-graphs differ topologically but they are non-disjunct; their intersection cannot be cut open and unfolded into a common, topology preserving band. A possible graph symbol for the combined **Ch** tubule in **Evl** is (4^6^8^6^);(6^2^). The (6^2^) part acts as a sort of exoskeleton since it prevents the (4^6^8^6^) from unfolding. The comparison of the topology of the **Ch** tubules in **Evl** with that of the silicate tubules in **Cha** itself [Figs. 6 ▸(*c*),  ▸6(*f*)] shows that the latter can be described as (4^3^8^3^) (therefore, the same as in **Evl** but with halved the identity period) and no external exoskeleton.

Similar to the **Cha** case, the circumference of the **Yk** tubule [Fig. 6 ▸(*a*)] can be constructed from four-, six- and eight-membered rings and given the symbol (4^12^6^8^8^4^) [Fig. 6 ▸(*d*)]. Unlike the case of the tubular chain discussed earlier, there are no outward building units, but complex inward ones. These are confined to two consecutive M-cubes, one occupied by a (Fe,Mn)O_5_ square pyramid and the adjacent ones being empty [Fig. 3 ▸(*i*)]. Each M-cube contains two horizontal [Si_2_O_7_]^6−^ groups crossing the nanorod axis. Searching for rings in the nodal representation via visual inspection led to around 37 different rings with orders 1 [for (Fe,Mn)–O_apical_ (or H_2_O)], 3, 4, 7, 8, 10 or 12, thus practically excluding an easy and intelligible graph representation for this partial structural unit and for the nanorod in its entirety.

The analysis of the topologies of the bands that serve as flat 1D prototypes for the tubules in **Evl** indicates that both can be considered as a combination of 1D bands cut out from the two 2D topologies shown in Fig. 6 ▸(*g*) and H. These topologies are specific for the crystal structures of the layer- and framework-based minerals that occur in association with **Evl**. In particular, the topology shown in Fig. 6 ▸(*g*) describes the topology of the {(TiO)[Si_2_O_7_]}^4−^ layer in the crystal structure of lamprophyllite, (SrNa)Ti_2_Na_3_Ti(Si_2_O_7_)_2_O_2_(OH)_2_ (Krivovichev *et al.*, 2003 ▸), which was found in association with **Evl**. In fact, we have observed in our sample a needle of **Evl** emanating from a small brownish-yellow plate of lamprophyllite. The topology shown in Fig. 6 ▸(*h*) serves as an underlying band for the tubules in **Cha** and (partially) for the **Ch** tubules in **Evl**. The [Si_2_O_5_]^2−^ layers of this topology occur in the crystal structures of many minerals, including those found in alkaline massifs. For instance, single layers of this topology are present in the crystal structures of the Khibiny minerals shlykovite and cryptophyllite (Pekov *et al.*, 2009 ▸), whereas double layers have been observed in delhayelite, hydro­delhayelite and fivegite, minerals that have been either observed or discovered in the Khibiny massif (Pekov *et al.*, 2010 ▸; Pekov *et al.*, 2011 ▸; Pekov *et al.*, 2012 ▸). In addition, the layers of this topology are parts of the feldspar topology and we note that K-feldspar occurs in association with both **Yks** and **Evl**. The topological similarity and close relationships between the 2D structures of associated minerals and the nanoscale tubules in **Evl** suggest possible structural links between layered silicate architectures and nanotubular motifs. One possible interpretation is that the nanotubules formed through transformation processes involving pre-existing layered silicates. Such transformations bear a resemblance to exfoliation and subsequent scrolling-based routes for the artificial fabrication of inorganic nanotubes under hydro­thermal conditions (Smith *et al.*, 2011 ▸). In some aspects, this scenario is similar to the production of carbon nanotubes from graphene layers that, in some particular cases, results in the formation of microcrystals consisting of carbon nanotubes (Schlittler *et al.*, 2001 ▸). It is quite probable that a similar process is at work in natural mineral systems. However, the available data do not provide direct evidence for such a mechanism in the case of **Evl**. An alternative possibility is that 2D structural fragments formed directly in hydro­thermal (or colloidal) solutions and subsequently curled into nanotubules that later assembled into a periodic three-dimensional framework over geological timescales. The two scenarios differ in the source of the 2D prototypical units for the formation of nanotubules that can be formed either from primary minerals during their exfoliation or in secondary solutions at the last stage of hydro­thermal activity. At present, both scenarios remain speculative.

Information-based complexity calculations show that **Evl** belongs to the class of very complex minerals, being on the seventh place in complexity among ∼6000 mineral species known so far (Krivovichev, 2013 ▸; Krivovichev *et al.*, 2022 ▸). Table 4 ▸ provides some crystallographic and complexity parameters for **Cha**, **Dnv**, **Yks** and **Evl**. The extreme complexity of **Evl** is of double origin: first, due to its binary nanotubule-based character (the presence of two different nanoscale tubules in the same structure) and, second, due to the formation of double superstructure along the *a* axis (direction of the tubule extension), induced by atomic ordering inside the **Yk** tubules (see above).

Finally, it is noteworthy that the mineral species listed in Table 4 ▸ as well as some other very complex silicate minerals discovered in alkaline massifs (Zolotarev *et al.*, 2020 ▸) have no synthetic analogs. The example of **Evl** shows not only that mineralogy has an enormous potential to discover structures unprecedented among the currently known synthetic materials but also to serve as an inspiration for the fabrication of novel materials with unique structures and possibly functional properties (Bindi *et al.*, 2020 ▸).

## 4. Materials and methods

### 4.1. Experimental design

The experiments performed in this paper were aimed at solving the structure of eveslogite, one of the most complex inorganic compounds on Earth. Because of the small size of the crystals and their intricate twinning, X-ray diffraction methods were not applicable. Instead, 3D ED was used for the data collection (Gemmi *et al.*, 2019 ▸). With this method, it was possible to avoid the twinning and to acquire data from single nm-sized crystals. HAADF-STEM was used to verify the structure and EDS was used to gain information about the elemental composition.

#### 4.2. Materials

The material used in this study was from the original holotype specimen found by Men’shikov *et al.* (2003 ▸) in the Khibiny alkaline massif. A sample of eveslogite was donated to W. Depmeier by I. V. Rozhdestvenskaya, who had received it from V. N. Yakovenchuk. This sample was then given to U. Kolb and E. Buchsteiner. Around the same time, an independent sample (also from V. N. Yakovenchuk) was handed over by S. V. Krivovichev to M. Klementova and L. Palatinus. In 2020 the two groups decided to combine their forces and efforts to pursue a common goal. The eveslogite crystals are light brown in color and have a fibrous morphology. For powdered samples, some fibers were mortared, suspended in iso­propanol and sprayed onto a carbon coated Cu grid with the help of an ultrasonic device. Additionally, three thin-foil samples were prepared via focused ion beam microscopy (FIB).

#### 4.3. Methods

At JGU Mainz, transmission electron microscopy (TEM) investigations were performed with a FEI Tecnai F30 (Eindhoven, Netherlands) (300 kV). A 4k × 4k CCD camera [Ultrascan 400 from Gatan (Pleasanton, CA, USA)] and a tomography sample holder from E. A. Fischione Instruments Inc. (Export, PA, USA) were used. Electron beam precession was applied via DigiSTAR from NanoMEGAS (Brussels, Belgium).

For 3D ED measurements, the method of automated diffraction tomography (ADT) (Mugnaioli *et al.*, 2009 ▸) was applied by using a Fast-ADT measurement routine in STEM mode (Plana-Ruiz *et al.*, 2020 ▸). ADT was performed in nano-beam mode, which allowed for a quasi-parallel beam setting up to 20 nm. In order to acquire an ADT dataset, a tracking file of the crystal was recorded, serving as reference images. Then the electron beam was placed at the region of interest with a size between 50 and 200 nm and a tilt series of diffraction patterns was recorded in 1° tilt steps. In order to reduce dynamical effects, a precession angle of 1° was used. In this case the datasets were recorded with a tilt range of −60° to +60°, a beam size of 120 nm, a camera length of 1 m and an exposure time of one second per frame.

At FZU Prague, 3D ED measurements were performed on an FEI Tecnai G2 20 (LaB6, 200 kV) equipped with a NanoMEGAS precession unit DigiSTAR, an Olympus SIS CCD camera Veleta (2048 × 2048px), and an EDAX windowless EDX detector Apollo XLTW. The sample was crushed under ethanol in an agate mortar. A drop of very dilute suspension was placed on a holey-carbon-coated Cu grid and allowed to dry by evaporation at ambient temperature.

The 3D ED data were collected by means of precession electron diffraction tomography (PEDT) (Kolb *et al.*, 2007 ▸; Kolb *et al.*, 2008 ▸) using a cooling holder at −177°C. About 40 crystals were screened for twinning by fast scan with a tilt step of 5° from −20 to +20°. Crystals without twinning (or with unequal twin individuals) were selected for detailed data collection. Each crystal was sequentially tilted by the step of 0.5° from −60° to +60° (depending on the crystal position on the grid the tilt range was slightly different for every crystal), and at every tilt step a precession diffraction pattern in microdiffraction mode was acquired using a precession angle of 0.7°.

The data processing was carried out in the *eADT* (Kolb *et al.*, 2012 ▸) and *PETS2* software (Palatinus *et al.*, 2019 ▸), including the correction of optical distortions (Brázda *et al.*, 2022 ▸). Datasets from two crystals with a slightly different orientation were merged to increase the data completeness (Fig. 1 ▸). Structure solution and kinematical refinement were performed in the computing system *JANA2020* (Petříček *et al.*, 2023 ▸). The structure was solved by the charge flipping algorithm using the program *SUPERFLIP* (Palatinus & Chapuis, 2007 ▸).

HRTEM was carried out on an FEI Tecnai TF20 X-twin microscope operated at 200 kV (FEG, 1.9 Å point resolution) equipped with an EDAX energy-dispersive X-ray (EDX) detector. HRTEM images were recorded on a Gatan UltraScan CCD camera with resolution of 2048 × 2048 pixels using the *Digital Micrograph* software package.

Chemical composition was measured on the monomineral bulk sample as well as on individual microcrystals using EDS. The following setups were used:

(1) an FEI Tecnai TF20 X-twin microscope operated at 200 kV (FEG, 1.9 Å point resolution) equipped with an EDAX energy-dispersive X-ray detector. The EDX spectra were processed using the FEI *TIA* software. Ten crystals were measured.

(2) an FEI Tecnai G2 20 (LaB6, 200 kV) equipped with an EDAX windowless EDX detector Apollo XLTW. The EDX spectra were processed using the EDAX *TEAM* software. Twelve crystals were measured.

(3) an electron probe microanalyzer Jeol JXA-8230 equipped with energy-dispersive spectrometer Bruker QUANTAX 200. Sample composition was measured at 20 kV by standardless quantification method P/B-ZAF. The software *Esprit 2* (Bruker) was used for data processing. Seven points were measured on the monomineral bulk sample.

For powder X-ray diffraction experiment (pXRD), the sample was ground and placed in the 0.5 mm borosilicate glass capillary. Powder diffraction data was collected using the Debye–Scherrer transmission configuration on the Empyrean (PANalytical) powder diffractometer (λCu *K*α = 1.54184 Å, 45 kV, 40 mA) that was equipped by a focusing mirror, capillary holder, PIXcel3D detector and an Oxford Cryostream cooling head 700 plus. The measurement was performed at a constant temperature of 100 K from 2θ = 3° to 2θ = 80° with a 0.013° step with a total measurement time of 3 h.

HAADF STEM images were taken using a FEI Titan 80-300ST equipped with an imaging corrector and a Fischione HAADF detector. The machine was operated at 300 kV and a beam current of about 6 pA during all experiments. Image simulations were performed using the *StemSim* code (Rosenauer & Schowalter, 2008 ▸) applying the parameters of the experiment.

The complexity of crystal structures was quantitatively estimated using Shannon information theory-based approach followed the procedures proposed by Krivovichev (2012 ▸) and Krivovichev (2014 ▸). The complexity was estimated as the amounts of Shannon information in bit per atom and per unit cell. The calculations have been performed using the *TOPOS Pro* software package (Blatov *et al.*, 2014 ▸).

## Supplementary Material

Crystal structure: contains datablock(s) global, I. DOI: 10.1107/S2052520626008620/je5066sup1.cif

Structure factors: contains datablock(s) I. DOI: 10.1107/S2052520626008620/je5066Isup2.hkl

Figs S1 and S2, Table S1. DOI: 10.1107/S2052520626008620/je5066sup3.pdf

CCDC reference: 2582316

## Figures and Tables

**Figure 1:**
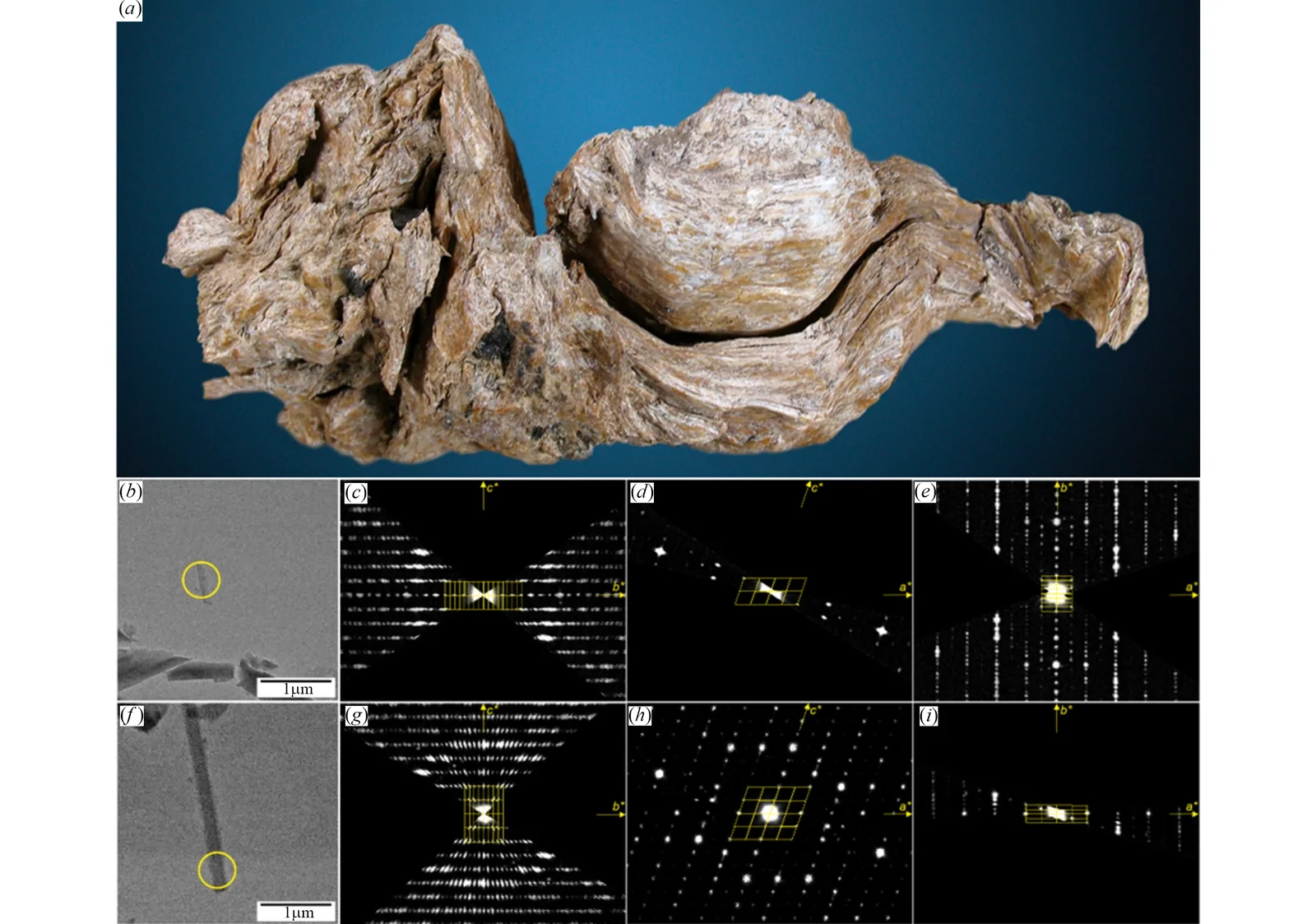
(*a*) A fibrous aggregate of mineral **Evl** from a monomineral vein in gneissose rischorrites of Eveslogchorr mountain. The length of the sample is 10 cm. Data from two untwinned crystals (*b* and *f*) were combined for the structure solution to increase the data completeness. Images of crystals and the sections through the diffraction data are shown: (*b*) crystal 1, (*c*) 0*kl* section, (*d*) *h*0*l* section, (*e*) *hk*0 section, (*f*) crystal 2, (*g*) 0*kl* section, (*h*) *h*0*l* section, (*i*) *hk*0 section. The grid of the unit cell is indicated in yellow.

**Figure 2:**
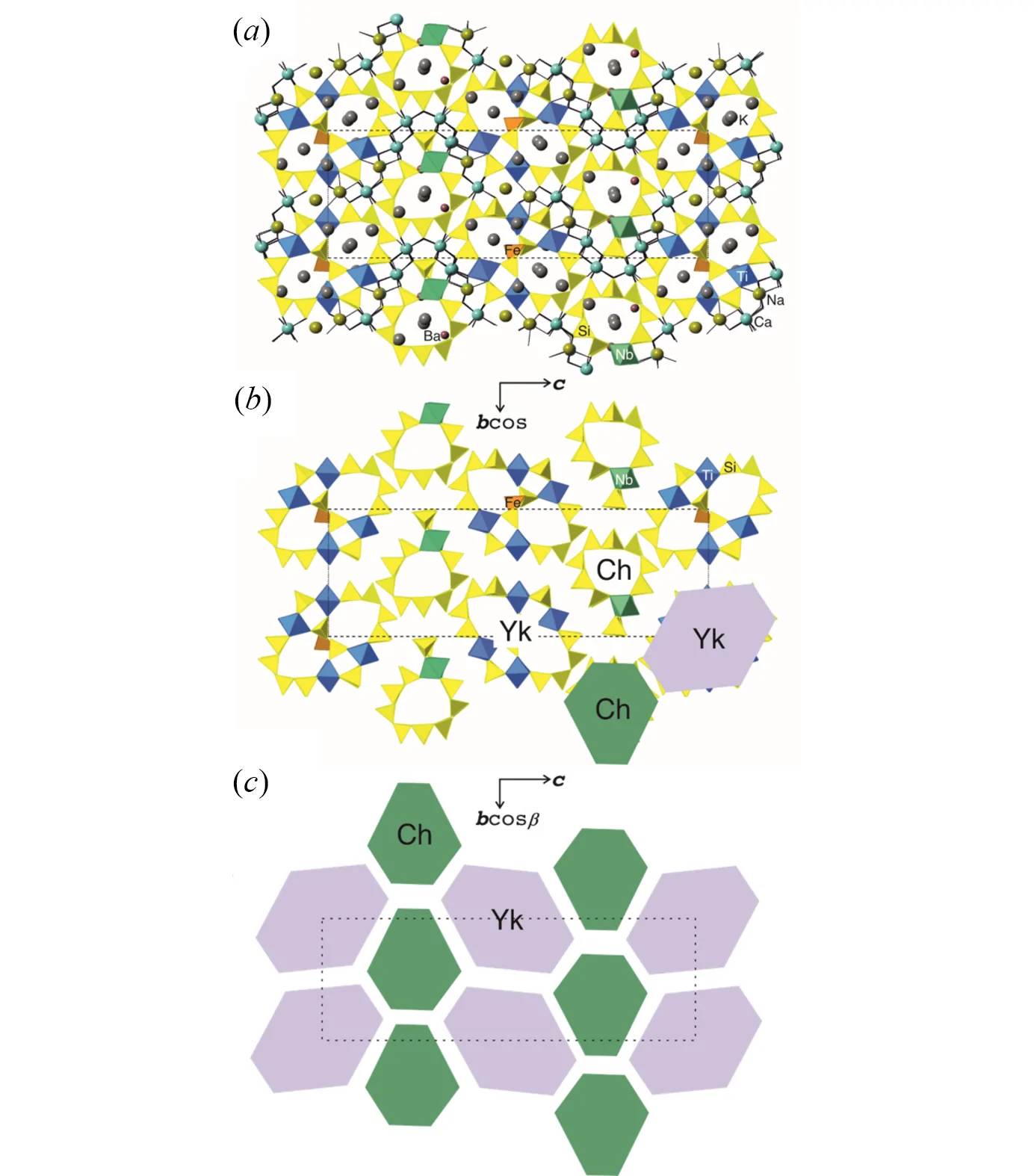
The crystal structure of eveslogite (**Evl**). (*a*) The projection of the full crystal structure along the *a* axis. (*b*) The projection of the crystal structure along the *a* axis showing only the tubules in polyhedral representation. (*c*) The scheme illustrating packing of the tubules.

**Figure 3:**
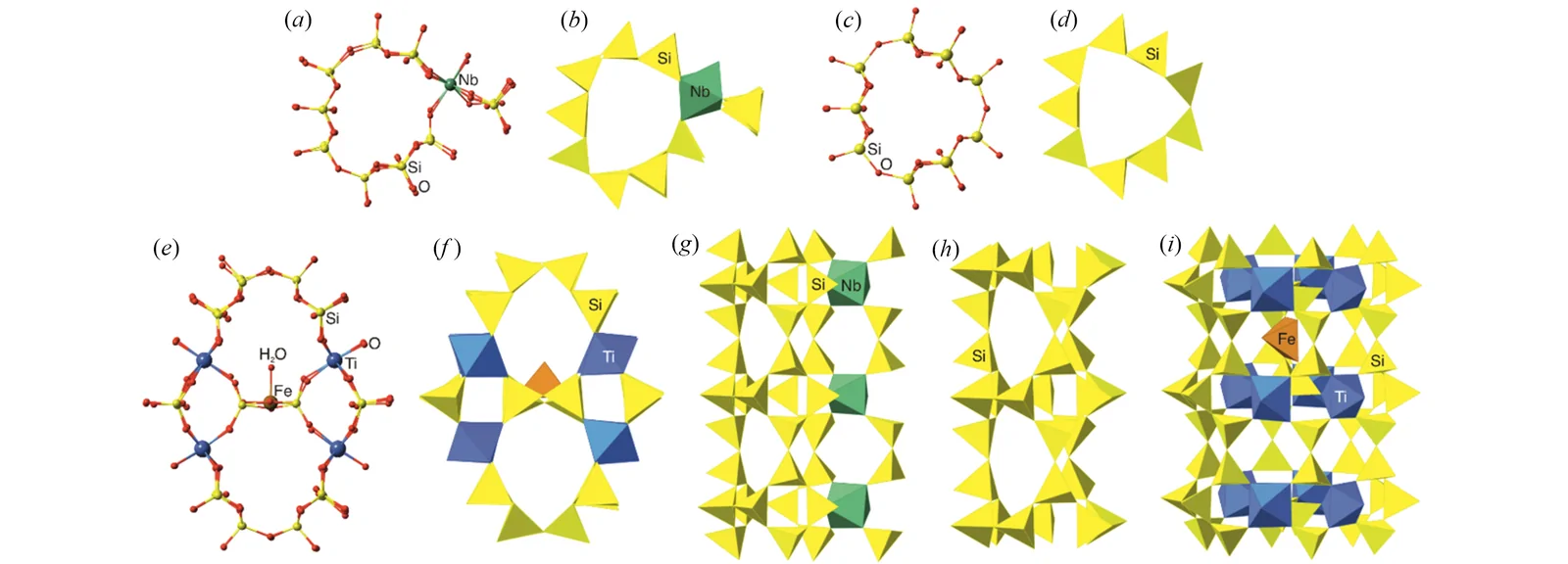
Nanoscopic tubular building units in eveslogite (**Evl**) compared to charoite (**Cha**). (*a*) Cross section of the charoite-like tubule (**Ch**) in **Evl** shown in ball-and-stick representation. (*b*) Cross section of the **Ch** tubule in **Evl** shown in polyhedral representation. (*c*) Cross section of the tubule in **Cha** shown in ball-and-stick representation. (*d*) Cross section of the tubule in **Cha** shown in polyhedral representation. (*e*) Cross section of the yuksporite-like tubule (**Yk**) in **Evl** shown in ball-and-stick representation. (*f*) Cross section of the **Yk** tubule in **Evl** shown in polyhedral representation. (*g*, *h*, *i*) Side views of the **Ch** tubule in **Evl**, **Ch** tubule in **Cha**, and **Yk** tubule in **Evl**, respectively.

**Figure 4:**
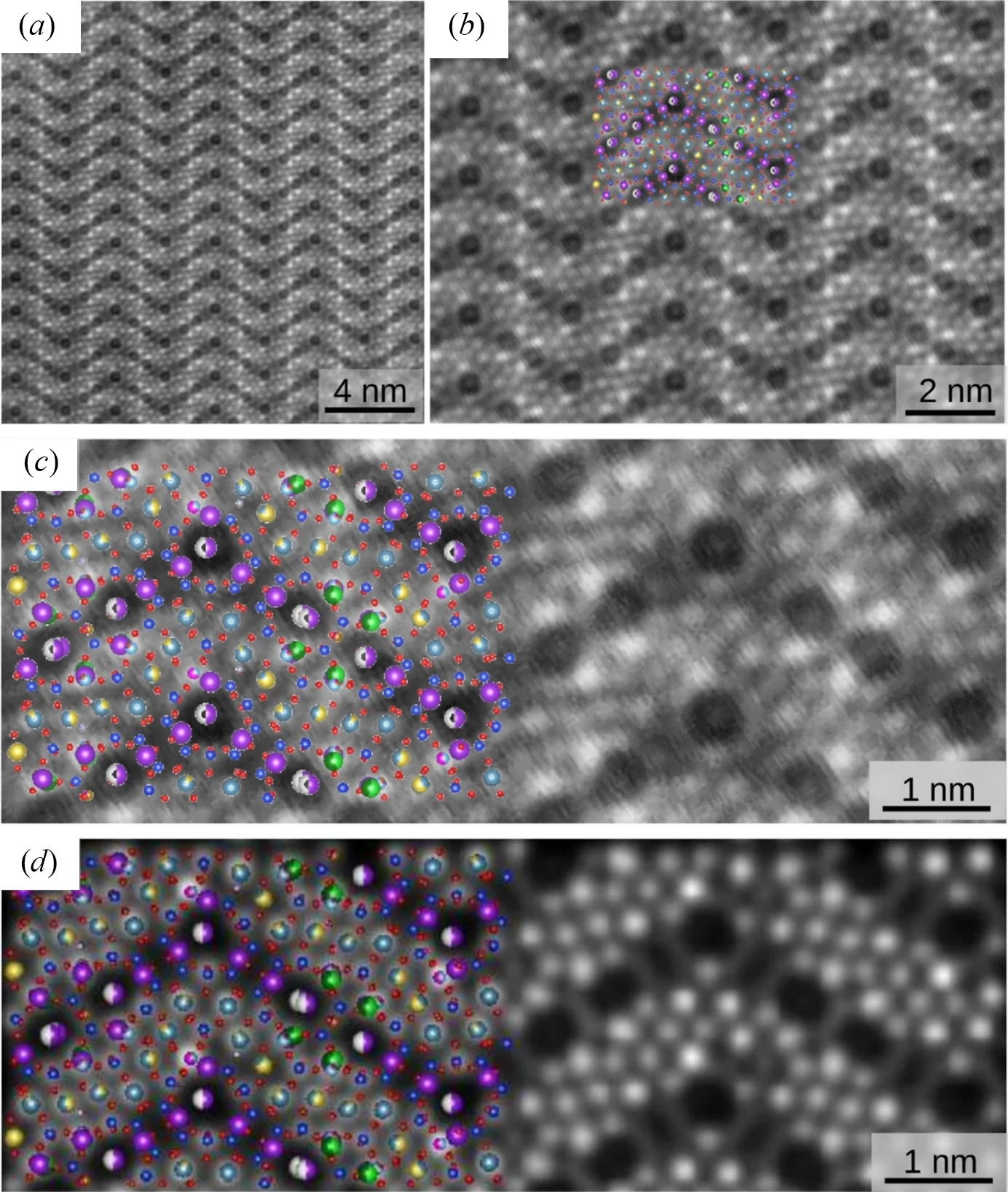
HAADF image analysis of the eveslogite (**Evl**) structure. (*a*) Filtered HAADF STEM image of **Evl** along the *a* axis. (*b*) and (*c*) Zoomed in view overlayed with the structure model of **Evl**. The atom positions match nicely. (*d*) Simulated HAADF image based on the determined **Evl** structure. Si is displayed in dark blue, K in purple, Ba in light green, Sr in dark green, Ca in blue-gray, Na in yellow, Ti in light blue, Nb in pink, Fe in orange, Mn in magenta, O in red, F in gray and H in white.

**Figure 5:**
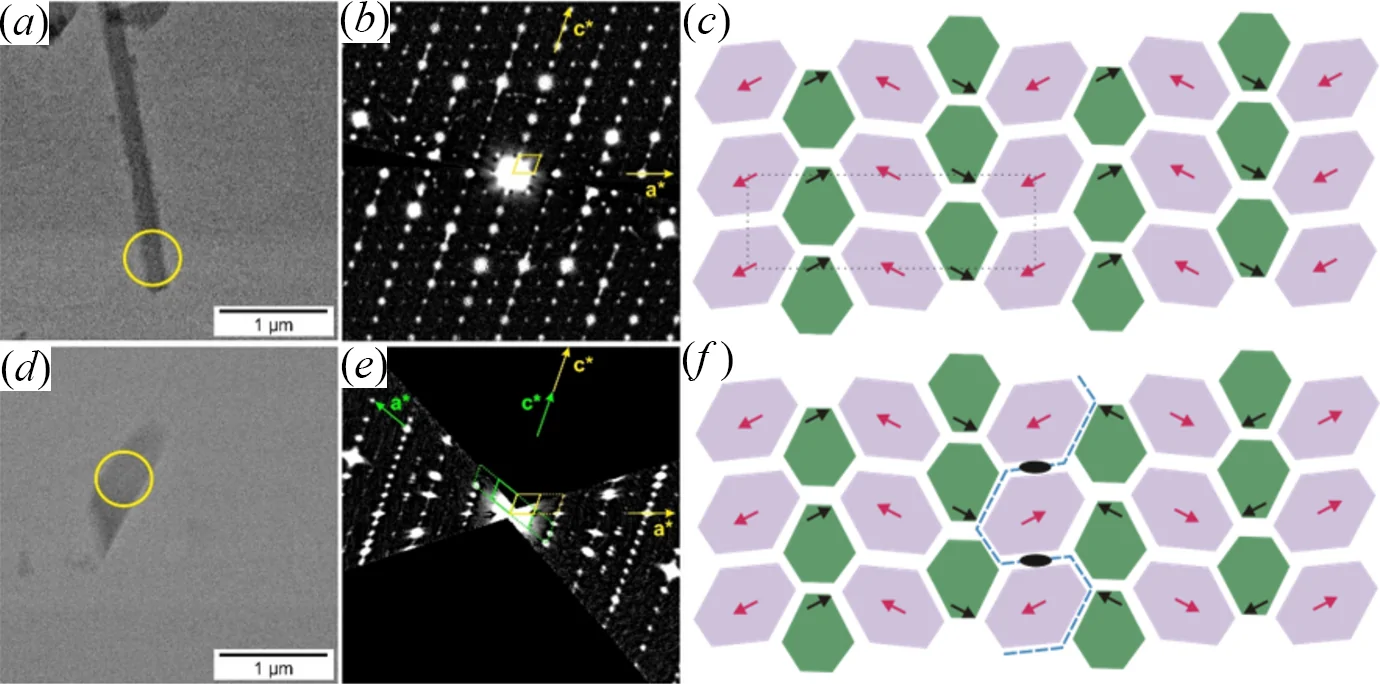
Twinning in **Evl**. TEM images of crystals and sections through diffraction data are shown: (*a*) untwinned crystal, (*b*) *h*0*l* section of an untwinned crystal. (*c*) Schematic projection of the crystal structure of an untwinned crystal of **Evl**. (*d*) Twinned crystal, (*e*) *h*0*l* section of a twinned crystal. (*f*) The scheme of twinning of **Evl** with two twin components related by a twofold rotation around the *a* axis. The red and black arrows show the orientations of the Fe–H_2_O and Nb–O bonds, respectively; the black ellipse indicates a twofold axis acting as a twin operator; the blue dashed line shows the border between different twin domains related by the twofold axis parallel to the *a* axis.

**Figure 6:**
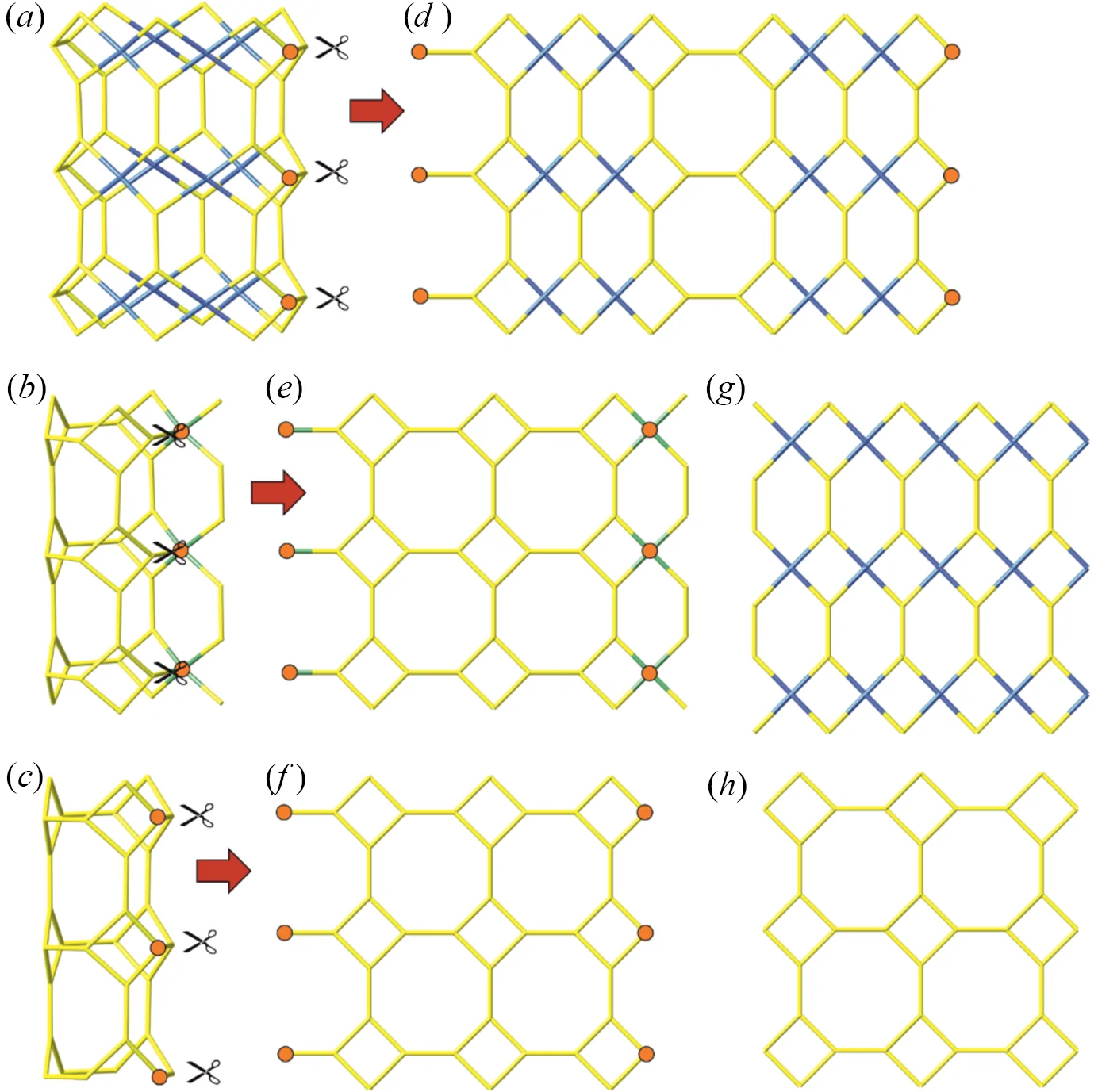
Topological analysis of the nanoscopic tubules in eveslogite (**Evl**) and charoite (**Cha**) by means of a nodal representation. The topologies of the yuksporite-like tubule (**Yk**) (*a*) and charoite-like tubule (**Ch**) (*b*) in **Evl**, as well as the tubule in **Cha** (*c*). (*d*, *e*, *f*) The bands corresponding to the topologies shown in (*a*, *b*, *c*), respectively. (*g*) The topology of the titanosilicate sheet in lamprophyllite. (*h*) The topology of the silicate sheet in apophyllite and feldspar-group minerals. The scissors indicate the edges that have to be cut in order to transform tubules into bands. The orange circles indicate points that have to be glued together in order to transform bands back to the tubules.

**Table 1:** Crystallographic information about eveslogite and information about the structure analysis The reflection count refers to merged reflections with observed reflections having *I*/σ > 3. The reflection/parameter ratio is based on all reflections

Sum formula	K_17.5_(Ba,Sr)_4_(Na,Ca)_40_[(Ti,Nb,Fe,Mn)_11_Si_62_O_179_(OH,F)_12_(O,OH)_13_](H_2_O)		
Composition	K_17.5_Ba_1.848_Sr_2.152_Na_11.909_Ca_28.091_Ti_6.7_Nb_1.6_Fe_1.7_Mn_1.0_Si_62_O_200_F_5_H_*n*_		
*a* (Å)	14.2359	Kinematical refinement	
*b* (Å)	44.8242	No. of reflections	54790 = 30466 + 24324
*c* (Å)	15.9058	Reflection/parameter ratio	51.45
α (°)	90	GOF(obs) (%)	3.23
β (°)	109.658	GOF(all) (%)	2.58
γ (°)	90	*R*(obs) (%)	19.91
*Z*	2	*R*(all) (%)	24.99
Unit-cell volume (Å^3^)	9558.079744	*wR*(obs) (%)	27.33
Space group	*P*2_1_	*wR*(all) (%)	28.54
Density (g cm^−3^)	2.8435		

**Table 2:** Overview of refined, constrained, restrained and inferred parameters in the **Evl** structure model

Structural parameter	Treatment	Structural parameter	Treatment
Unit-cell parameters	Refined from powder diffraction data	Ti/Nb/Fe/Mn occupancies in Yk tubule	Fixed based on average composition
Space group	Determined via 3D ED	(Fe,Mn)O_5_ occupancy	Fixed based on average composition
Atomic coordinates	Refined	Si–O distances	Restrained to 1.61 Å (σ = 0.01 Å)
Ba/Sr occupancies	Refined	H/OH/F locations	Not refined, based on crystallo-chemical analysis and charge balance
Ca/Na occupancies	Refined	*U*_iso_ values	Refined isotropically with one common *U*_iso_ value per atom species
Ti/Nb occupancies in **Ch** tubule	Refined		

**Table 3:** Chemical composition of eveslogite (in atoms per formula units, apfu) normalized to 62 Si atoms in the structure Oxygen is assigned by stoichiometry and hydrogen was not measured.

Element		Ca	Na	K	Ba	Sr	Ti	Nb	Fe	Mn	Si	F	Cl
Range	min	24.1	10.0	12.3	0.9	2.0	5.0	0.7	1.3	0.8	62	0	0
	max	37.1	22.1	21.1	3.3	3.4	8.5	3.3	2.5	1.5		11.3	2.4
Average		28.5	13.3	15.6	2.0	2.6	6.7	1.6	1.7	1.1	62	3.0	1.0

**Table 4:** Crystallographic and structural complexity parameters for minerals based on nanotubules The unit-cell parameter along the tubule extension is given in bold.

Mineral name	Charoite		Denisovite	Yuksporite	Eveslogite
Chemical formula†	(K,Sr,Ba,Mn)_15-16_(Ca,Na)_32_[Si_70_(O,OH)_180_](OH,F)_4_·*n*H_2_O		K_14+*x*_(Ca,Na,Mn,Fe)_48_[Si_60_O_162_]F_16_(O_*x*_,OH_4–*x*_)·2H_2_O	K_4_(Ca,Na)_14_(Sr,Ba)_2_(Mn,Fe)(Ti,Nb)_4_(O,OH)_4_(Si_6_O_17_)_2_(Si_2_O_7_)_3_(H_2_O,OH)_3_	K_17.5_(Ba,Sr)_4_(Na,Ca)_40_[(Ti,Nb,Fe,Mn)_11_Si_62_O_179_(OH,F)_12_(O,OH)_13_](H_2_O)
Space group	*P*2_1_/*m*		*P*2_1_/*a*	*P*2_1_/*m*	*P*2_1_
*a* (Å)	31.96	32.11	31.024	**7.126**	**14.24**
*b* (Å)	19.64	19.77	19.554	24.913	44.82
*c* (Å)	**7.09**	**7.23**	**7.1441**	17.075	15.91
β (°)	90.0	95.85	95.99	101.89	109.66
*V* (Å^3^)	4450	4566	4310	2966	9558
*I_G_* (bit/atom)	6.502		6.392	5.944	8.480
*I*_*G*,total_ (bit/atom)	2119.685		2083.685	1379.052	6054.453

† The chemical formula approved by the International Mineralogical Association (IMA).

## Data Availability

All data are available in the main text or supplementary materials. Raw data can be provided upon request.
