# Supplementary material for: Crystal structure of eveslogite revealed by electron diffraction techniques: a titanosilicate mineral with structurally and compositionally distinct nanoscopic tubular units
Source: Acta Crystallogr B Struct Sci Cryst Eng Mater. 2026 Sep 1;82(Pt 5):500–12. doi: 10.1107/S2052520626008620 (PMC13729042; doi:10.1107/S2052520626008620)
Supplement: Supplementary file 1 [file b-82-00500-sup3.pdf]

STRUCTURAL SCIENCE
CRYSTAL ENGINEERING
MATERIALS

Volume 82 (2026)

Supporting information for article:

The crystal structure of eveslogite revealed by electron diffraction techniques: a titanosilicate mineral with structurally and compositionally distinct nanoscopic tubular units

Emilia Buchsteiner, Mariana Klementová, Wulf Depmeier, Sergey V. Krivovichev, Michael Czank, Marco Schowalter, Lukas Palatinus, Ira V. Rozhdestvenskaya, Viktor Yakovenchuk and Ute Kolb

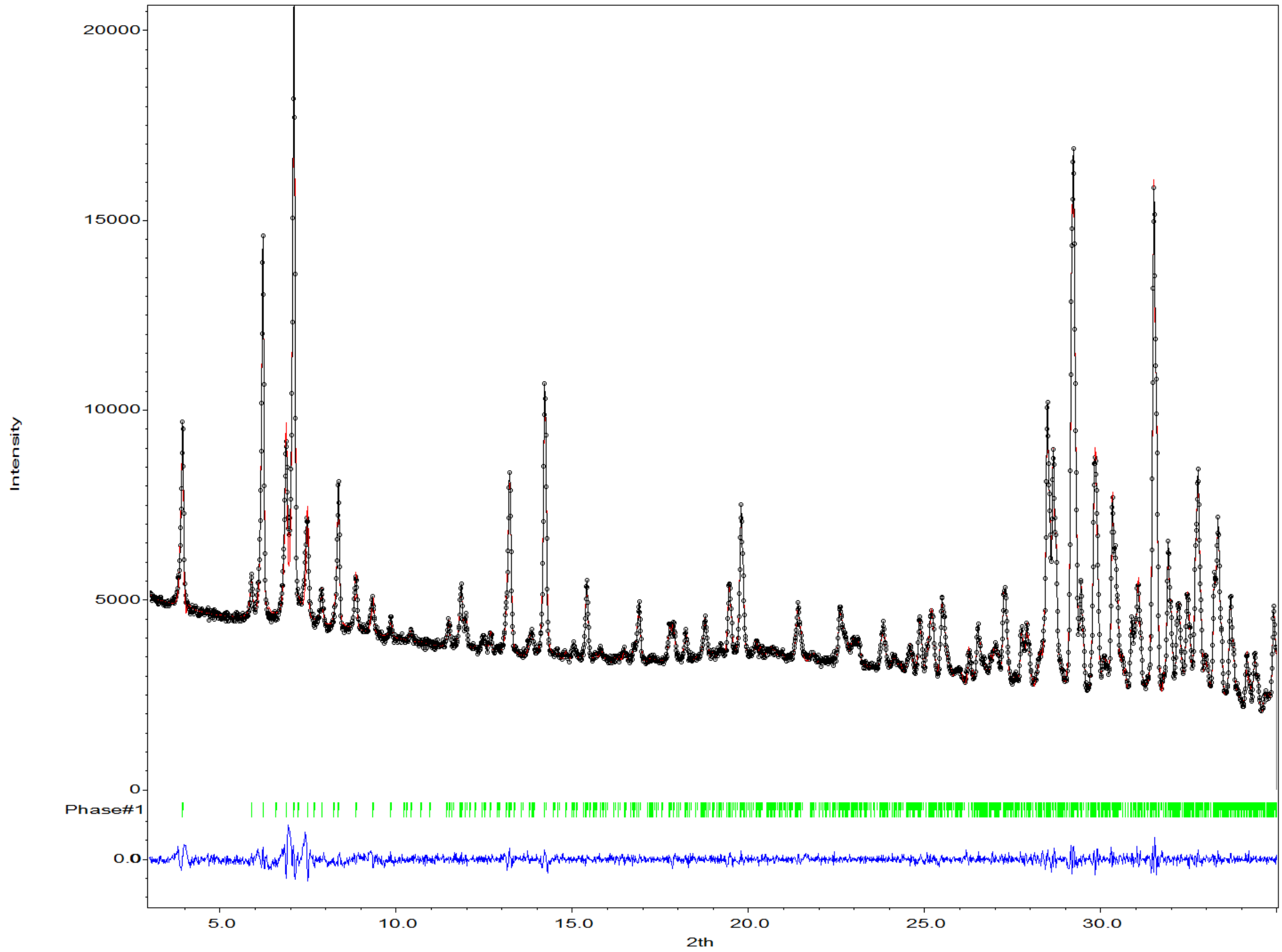

Figure S1 Powder XRD data of eveslogite (**Evl**). LeBail fit from 2θ = 3° to 2θ = 35° resulting in GOF = 1.48, Rp = 1.69 and wRp = 2.27.

Table S1 List of possible hydrogen bonds based on bond valence calculations with JANA2020. The maximum donor-hydrogen bond distance was set to 1.4 Å and the maximum hydrogen-acceptor bond distance to 2.5 Å.

Donor	Acceptor	D-H distance	H…A distance	D-A distance	A-H…D angle
O102	O142	0.96	2.41	3.113(18)	130.23
O109	O174	0.96	2.37	3.105(18)	133.32
O51	O66#t-1,0,0	0.96	2.03	2.595(13)	115.79
O51	O71#t-1,0,0	0.96	2.28	3.209(13)	162.17
O67	O58	0.96	2.28	3.201(13)	159.86
O67	O92	0.96	1.87	2.523(14)	122.45
O71	O51#t1,0,0	0.96	2.33	3.209(13)	151.55
O71	O66	0.96	2.50	2.678(10)	90.15
O11	O151	0.96	2.23	2.680(16)	107.20
O13	O150	0.96	2.25	2.709(16)	108.44
O58	O67	0.96	2.37	3.201(13)	144.06
O74	O54#t-1,0,-1	0.96	2.20	2.714(13)	112.28
O94	O9	0.96	2.23	2.646(13)	105.31
O101	O22	0.96	2.37	2.707(13)	100.06
O117	O86	0.96	2.15	2.605(13)	107.59
O93	O204#s2t1,0,2	0.96	2.31	3.251(14)	167.52
O96	O87#t-1,0,0	0.96	2.17	3.125(18)	176.36
O106	O196	0.96	2.20	3.153(13)	171.71
O147	O42	0.96	2.48	3.060(17)	118.55
O147	O122	0.96	2.20	3.155(13)	176.01
O188	O107	0.96	2.10	3.052(13)	173.44

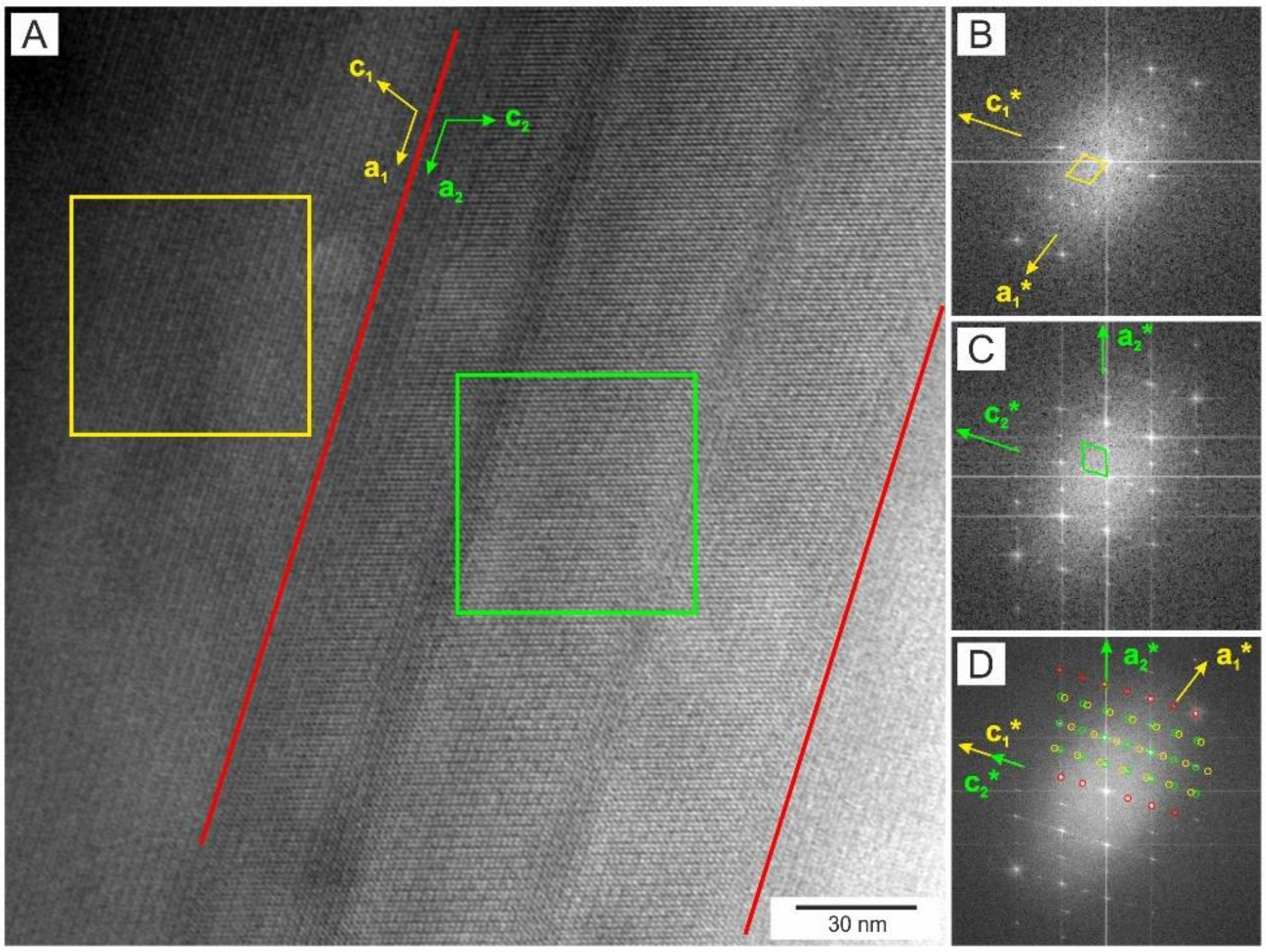

Figure S2 Twinning in eveslogite (**Evl**). (**A**) HRTEM image of a twinned **Evl** crystal with squares showing the areas of fast-Fourier-transforms (FFTs) of the two domains separated by red lines. (**B**) FFT of the yellow square corresponding to twin domain 1, (**C**) FFT of the green square corresponding to twin domain 2, (**D**) FFT of the whole image showing the twinning relationship of both domains.
